# Near optimal graphene terahertz non-reciprocal isolator

**DOI:** 10.1038/ncomms11216

**Published:** 2016-04-06

**Authors:** Michele Tamagnone, Clara Moldovan, Jean-Marie Poumirol, Alexey B. Kuzmenko, Adrian M. Ionescu, Juan R. Mosig, Julien Perruisseau-Carrier

**Affiliations:** 1 Laboratory of Electromagnetics and Antennas (LEMA), Ecole Polytechnique Fédérale de Lausanne, EPFL, Station 11, 1015 Lausanne, Switzerland; 2 Nanoelectronic Devices Laboratory (NANOLAB), École Polytechnique Fédérale de Lausanne, EPFL, Station 11, 1015 Lausanne, Switzerland; 3 Department of Quantum Matter Physics (DQMP), Université de Genève, Quai Ernest-Ansermet 24, 1211 Genève-4, Switzerland; 4 Adaptive MicroNano Wave Systems, Ecole Polytechnique Fédérale de Lausanne, EPFL, Station 11, 1015 Lausanne, Switzerland

## Abstract

Isolators, or optical diodes, are devices enabling unidirectional light propagation by using non-reciprocal optical materials, namely materials able to break Lorentz reciprocity. The realization of isolators at terahertz frequencies is a very important open challenge made difficult by the intrinsically lossy propagation of terahertz radiation in current non-reciprocal materials. Here we report the design, fabrication and measurement of a terahertz non-reciprocal isolator for circularly polarized waves based on magnetostatically biased monolayer graphene, operating in reflection. The device exploits the non-reciprocal optical conductivity of graphene and, in spite of its simple design, it exhibits almost 20 dB of isolation and only 7.5 dB of insertion loss at 2.9 THz. Operation with linearly polarized light can be achieved using quarter-wave plates as polarization converters. These results demonstrate the superiority of graphene with respect to currently used terahertz non-reciprocal materials and pave the way to a novel class of optimal non-reciprocal devices.

## Abstract (web summary)

Optical isolators, or optical diodes, allow electromagnetic radiation to travel in one direction but not the other. Here, the authors achieve unidirectional propagation of terahertz waves by taking advantage of the non-reciprocal nature of optical conductivity in magnetostatically biased graphene.

## Graphical abstract

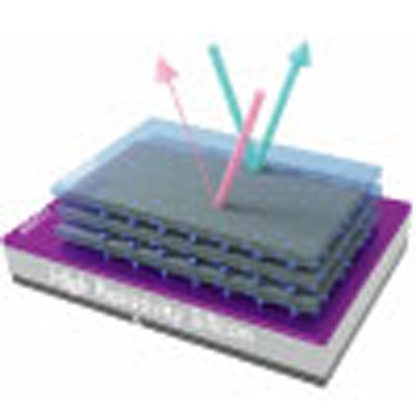

The potential applications of graphene for terahertz passive and active devices have received considerable attention in recent scientific literature^1^,^2^,^3^,^4^,^5^,^6^,^7^,^8^,^9^,^10^,^11^,^12^,^13^,^14^,^15^,^16^,^17^,^18^,^19^,^20^,^21^. One of the most promising research directions is the possibility of breaking the time-reversal symmetry in photonic devices based on magnetostatically biased graphene. The clearest evidence of such non-reciprocal effects is the experimental demonstration of Faraday rotation in graphene for terahertz frequencies^1^,^2^. Several theoretical works have proposed devices based on magnetostatically biased graphene^6^,^8^,^19^,^20^, including non-reciprocal isolators (also known as optical diodes), that is, photonic devices that support unidirectional light propagation. In addition, a narrowband graphene isolator was recently measured at 20 GHz (refs 5, 22). Ferrite isolators have been demonstrated in the THz range^23^, showing excellent operational bandwidth and eliminating the requirement for an external biasing magnetic field. However, currently available ferrite isolators are useful only up to 500 GHz and show prohibitive insertion losses in the order of tens of dB (ref. 23) beyond this frequency. This intrinsic limit is due to losses in ferrites, and motivates research in graphene and alternative materials. Precisely because of the losses in available magneto-optical materials, the realization of low loss non-reciprocal isolators is considered one of the most important challenges in terahertz science.

Apart from ferrite and graphene, alternative materials have also been proposed to achieve efficient terahertz non-reciprocity, and several works have been recently published presenting experimental characterization of the properties of these materials. One example is given by other free carrier based materials such as doped silicon^24^. In addition, thin films of HgTe exhibit interesting non-reciprocal properties due to a combination of band structure effects and high mobility carriers^25^. Ferrofluids have also been considered, since they exhibit good transparency in the THz band^26^. Another promising example are multiferroic materials, which show strong non-reciprocity and unidirectional propagation at terahertz frequencies^27^,^28^. However, these materials have not been employed for the experimental demonstration of final isolator designs, and rarely the explored frequencies exceed 1.5 THz.

In the following we aim to exploit the non-reciprocity of magnetically biased monolayer graphene using a reflection configuration to achieve isolation for circularly polarized waves. The concept of isolator for circularly polarized light has been presented theoretically in a transmission configuration for graphene and other magneto-optical materials^19^,^29^, while strong circular dichroism was predicted for similar reflection structures^20^. The device presented in this contribution achieves isolation for circularly polarized waves at 3 THz and 7.5 THz, showing performances very close to the theoretical upper bound for non-reciprocal graphene devices^4^ with almost 20 dB of isolation and 7.5 dB of insertion loss. Excellent agreement between simulations and measurements is also demonstrated.

## Results

### Design and fabrication

**Figure 1:**
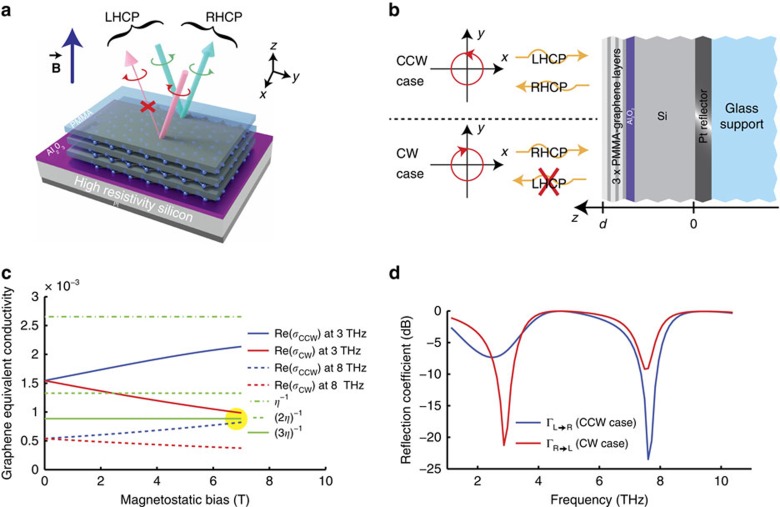
Structure and principle of operation of the proposed isolator. (**a**,**b**) 3D view and cross-section of the proposed isolator. The behaviour with respect to circularly polarized light is shown. Incident LHCP terahertz waves are reflected as RHCP by the isolator, while incident RHCP waves are absorbed. (**c**) Magnetic field induced splitting of *σ*_CW_ and *σ*_CCW_ as a function of the bias *B* (*f*=3 THz and 8 THz, *μ*_c_=0.53 eV, *τ*=35 fs, *T*=290 K) computed using the Kubo formula. The real part of the equivalent conductivity for the clockwise and counter-clockwise cases is shown for monolayer graphene and compared with multiples of the free space impedance *η*. In yellow is the area of interest for the design. (**d**) Simulation of the reflection coefficients for wave converted from right-handed to left-handed or vice-versa. Two working points are observed, however, the direction of the isolation in the second one is reversed. Reflection coefficients expressed in dB follow the rule 20 log_10_ Γ consistently with the definition of Γ in terms of reflected field amplitude.

A non-reciprocal isolator is a device possessing at least one electromagnetic mode showing unilateral propagation, that is, it allows the transmission of an electromagnetic wave in a given direction while strongly attenuating it in the other one (namely if time reversal transformation is applied to the wave)^30^. This definition is general and applies both to guided devices (that is, based on waveguides) and planar ones (that is, acting on plane waves or beams in free space). The proposed graphene terahertz isolator is planar, and it is illustrated in Fig. 1a,b (see also Supplementary Figs 1 and 2 and Supplementary Note 1 for fabrication process). A number *N* of graphene sheets are placed on a back-metallized thin silicon layer of 10 μm thickness (for our device *N*=3). The sheets are separated by thin poly methyl methacrylate (PMMA) layers (∼60 nm), while the thickness of the metallization (chromium and platinum) is 200 nm. The whole structure is bonded to a pyrex wafer, which has solely the function of mechanical support. A magnetostatic field *B* is applied orthogonally to graphene.

The device operation is based on reflecting incident left hand circularly polarized (LHCP) plane waves as right hand circularly polarized (RHCP) ones, while absorbing RHCP incident waves. The device thus achieves non-reciprocal unidirectional propagation and isolation for circularly polarized waves^19^,^23^,^24^ because time reversal transformation preserves the handedness of the propagating wave (for example, a time reversed LHCP is still LHCP). In addition, as explained later, simple reciprocal polarizers and polarization converters can be combined with this device to achieve terahertz isolation and source protection also for linearly polarized light.

Our device exploits Fabry–Perot resonances in the silicon layer to increase light-matter interaction in graphene. As a result, three monolayers of graphene are sufficient to obtain near perfect isolation. The principle of the isolator consists in creating for clockwise (CW) rotating waves (incident RHCP or reflected LHCP) a total surface impedance equal to the impedance *η* of free space (that is, impedance matching), causing total absorption (reflection coefficient 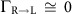). On the contrary, for counter-clockwise (CCW) ones (incident LHCP or reflected RHCP) the impedance is mismatched, and waves are reflected 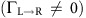. This phenomenon can be completely understood by solving Maxwell's equations in the structure, and it is due to the fact that graphene conductivity can be expressed as a scalar quantity for circular polarization (Fig. 1c), taking two different values *σ*_CW_ and *σ*_CCW_ in the CW and CCW cases, respectively. This fact can be easily understood considering that magnetostatically biased graphene exhibits the conductivity tensor^31^:


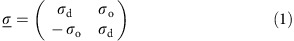


which has two eigenvalues *σ*_CW_=*σ*_d_+*jσ*_o_ and *σ*_CCW_=*σ*_d_−*jσ*_o_ for circularly polarized waves.

The yellow region in Fig. 1c shows that at 3 THz and for *B*=7 T the real part of *σ*_CW_ is very close to (3*η*)^−1^ and thus the total impedance of the three monolayers stack is very close to *η*. The role of the metallized silicon reflector is to cancel the imaginary part of *σ*_CW_ at the working frequency in order to enable full impedance matching in the CW case. Additional details can be found in the full design procedure provided in Supplementary Notes 2 and 3 and Supplementary Fig. 3. The simulated performances of the device are shown in Fig. 1d. Apart from the main working point at 2.9 THz, the device shows a second one at 7.6 THz where, however, the direction of the isolator is reversed (RHCP is reflected as LHCP and LHCP is absorbed). This inversion is explained by the different conductivity of graphene at higher frequency (Fig. 1c).

### Device measurement

**Figure 2:**
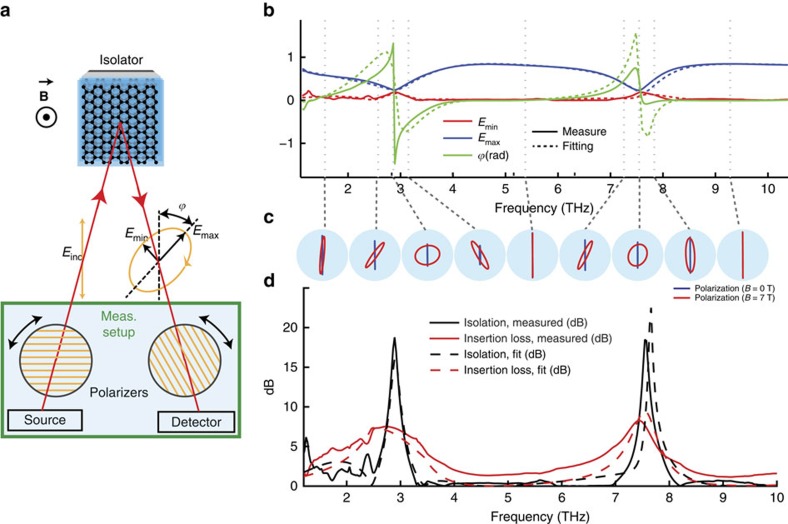
Measured isolator performances. Measures have been performed at *T*=290 K. (**a**) Schematic of the measurement setup configuration and definition of elliptical polarization parameters. (**b**) Measured elliptic polarization parameters (major and minor axis *E*_min_, *E*_max_ and Kerr rotation angle, *ϕ*) as a function of frequency for *B*=0 T and 7 T. *E*_min_, *E*_max_ are normalized with respect to *E*_inc_. The measures have been fitted (dashed traces) with the multilayer model (see Supplementary Note 4), and the best fit is obtained for *μ*_c_=0.53 eV, *τ*=35 fs, *d*=9.15 μm, additional loss: 30%. (**c**) Polarization state shown for some representative frequencies. (**d**) The extracted performances (isolation and insertion loss expressed both as positive dB quantities) of the isolator for circularly polarized waves. See also Supplementary Fig. 4.

The fabricated device has been measured using a Fourier transform infrared spectrometer connected to a split-coil superconducting magnet. A polarizer is used to create a linearly polarized incident light, while an analyser (that is, a second polarizer) is used in front of the detector (see Fig. 2a). The reflected elliptical polarization is mapped by repeating the measurement for different values of the angle *θ* between the two polarizers. The magnetostatic field is normal to the sample surface, while the light **k** vector is close to normal. Supplementary Fig. 4 shows that the reflected polarization is identical to the incident one for *B*=0 T. One can see that the normalized reflection shows strong absorption dips. These strong absorption features have a periodicity of 4.65 THz, corresponding to Fabry–Perot oscillation in a silicon layer of 9.43 μm in very good agreement with the nominal value. Figure 2b shows instead a rich polarization behaviour for *B*=7 T. This is also shown better by the polarization diagrams in Fig. 2c at selected frequencies of interest. At the first working point we also notice that the Kerr rotation *ϕ* goes up to 90° and continues from −90° to 0°.

The isolation (ISO) and insertion loss (IL) of the device are defined as:


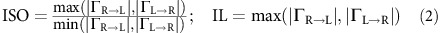


Even though our measurement setup is equipped only with linear polarizers, both these quantities can be computed accurately from the measured polarization parameters (obtained by exciting the device with a linear polarization and mapping the reflected polarization with the analyser). The sought isolation and insertion loss can then be retrieved as:


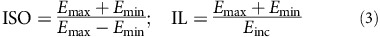


where, *E*_max_ and *E*_min_ are the major and minor axis of the mapped elliptical polarization of the reflected electric field and *E*_inc_ is the linearly polarized incident electric field. The full algorithm is actually more complex and it enables the compensation of polarizer imperfections (see Supplementary Note 4 for more details).

The resulting values are plotted in Fig. 2d. At both working frequencies the isolation reaches almost 20 dB (18.8 dB and 18.5 dB, respectively) and the insertion loss is ∼7.5 dB. The results plotted in Fig. 2b have been fitted with the layered model illustrated in the Supplementary Note 2, reaching a very good agreement. Conductivity of graphene is computed using the Kubo formula, and the best fit is obtained for a graphene Fermi level, *μ*_c_=0.53 eV, a graphene carrier scattering time, *τ*=35 fs, a silicon thickness, *d*=9.15 μm, and the fitting improves sensibly if an additional overall loss of 30% is added to the model over the whole bandwidth. The high *μ*_c_ can be explained considering that graphene is still in contact with the PMMA on one or two sides, and hence is likely to be highly doped by substrate interactions. The 30% loss could be attributed to a systematic error due to the non-perfect planarity of the isolator, which caused part of the energy to be reflected out of the detector.

**Figure 3:**
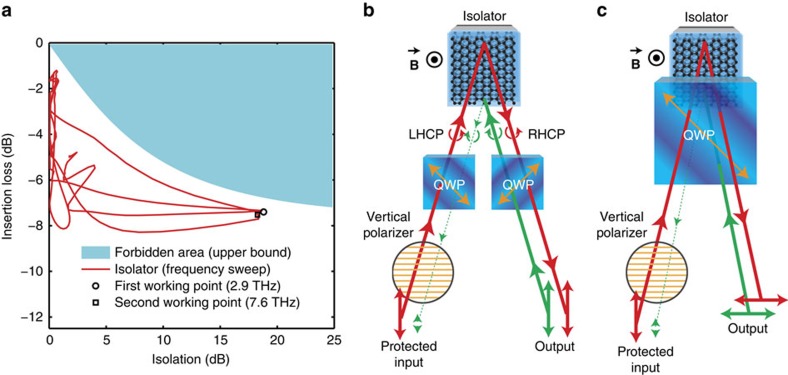
Isolator optimality and linear polarization schemes. (**a**) Representation of the measured device performances for *B*=7 T in the Cartesian plane between isolation and insertion loss for different frequencies. On the same plot the non-reciprocity theoretical upper-bound^4^ is represented for *γ*_NR_=0.295, value found from the fitted graphene parameters. The working frequencies (showing a maximum in the isolation) are highlighted. Because the curves are very close to the theoretical bound, the device is quasi-optimum with respect to it. (**b**,**c**) By combining the proposed isolator for circular polarization with simple QWPs it is possible to also achieve isolation for linearly polarized waves. The QWPs are placed with the optical axis (orange) at 45°, thus acting as polarization converters from circular to linear polarization. Two configurations are proposed. Polarizers and/or filters can be used to completely protect a source from cross polarization or other frequency signals coming from the output port.

Figure 3a is a Cartesian plot of the isolation versus the insertion loss. It has been shown that a theoretical upper bound exists in this plane for passive non-reciprocal isolators^4^, and that this bound can be expressed as a forbidden region whose size depends on the graphene parameters, the working frequency and the applied magnetic field. The bound inequality is:


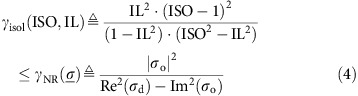


The right member *γ*_NR_ is a 2D material non-reciprocity figure of merit, which depends uniquely on the 2D conductivity tensor of graphene. High values of *γ*_NR_ correspond to less restrictive forbidden regions and hence lower insertion losses and better performances are possible; we also demonstrated that using the Drude–Lorentz approximation 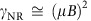, where *μ* is graphene mobility. The left term *γ*_isol_ (which is bounded) is a device figure of merit of the isolator, which depends on the isolation and on the insertion loss, and it defines the shape of the forbidden region. Notably, it forbids points having at the same time high isolation and low insertion loss, which represent the target for any isolator design.

For the fitted parameters, it can be shown that the forbidden region is frequency-independent in the band from 0 THz to 20  THz. The performance of the device is just 1 dB below the theoretical upper bound, which means that the device is near optimal.

While the presented isolator operates with circularly polarized light, it is clear that most terahertz applications need components able to handle linearly polarized waves. It is however, quite simple to adapt our isolator to linear polarization operation using one or two quarter-wave plates as polarization converters, as shown in Fig. 3b,c. This simple system can be used to protect a linearly polarized source from harmful reflections, which is one of the most important applications of non-reciprocal isolators^23^. The wave produced by the source could in fact propagate through the isolator but any reflected signal trying to propagate backwards in the isolator would be highly attenuated, protecting the source. If a linear polarizer is added after the source, then the latter is also protected from cross-polarized reflected light. Similarly, bandpass filters can be used in cascade with the isolator to protect the source from any unwanted signals having frequency outside the working band of the isolator. Supplementary Figs 4 and 5 show additional measurements performed on the device at various magnetic fields.

## Discussion

In this work we demonstrated the possibility of designing and implementing close to optimal terahertz isolators based on graphene. To achieve this goal, we proposed a reflection structure that exploits the Fabry–Perot resonances in a thin layer of silicon to obtain isolation using just three graphene monolayers. The operation of the device can be fully understood in the framework of Maxwell's equations using a 2D linear conductivity tensor to model carrier dynamics in magnetostatically biased graphene. One of the most significant aspects of this design is its ability to be very close to the optimal performances available with the used graphene^4^. Equivalent devices operating in transmission require more complicated structures or lead to suboptimal performances^4^,^5^,^19^.

In addition, it is worth mentioning some additional advantages related to this particular device geometry. First, due to the fact that the device is planar (that is, operating for plane waves and not based on mono-modal waveguide ports), it works for incident waves having different **k** vectors at the same time. Electric field effect in gated graphene could be used to fine tune the device to virtually infinite isolation, as demonstrated also at microwave frequencies^22^. In fact, perfect isolation is obtained when the device surface impedance is equal to free space impedance, and electrostatic gating allows a fine tuning of the total impedance of the device.

Because the device exploits Fabry–Perot resonances, it is relatively narrowband; however, the absolute bandwidth is in the order of 50 GHz, which is an excellent value for telecommunications and for continuous wave applications. This value can possibly be increased using more complex designs including patterned graphene, added metal patterns and more complex multilayer structures^32^. The relative precision of the working frequency is given by the substrate thickness, and hence it can be controlled and fine-tuned in a precise way, in the technological process, by polishing or using additive dielectric depositions. Furthermore, for different angles of incidence, the working frequency is expected to change due to the reduced longitudinal wavelength of the wave, and this could be used to dynamically tune the operation frequency.

Finally, the reflection configuration of the device eliminates completely the input impedance mismatch (return loss) issue, which is instead a concern for any device operating in a transmission configuration. For waves with a non-zero incidence angle (as the case for practical application where receiver and transmitter are in separate locations) this fact is evident considering that the device is invariant to translation and hence it operates with a single diffraction order. This implies that the wave cannot possibly be reflected to the receiver. For normally incident waves, this is due to the fact that an incident left hand wave can only be reflected as right hand and vice versa, because the device is invariant to rotation.

The main drawbacks of our device are the need of high magnetic field (7 T) and an insertion loss of more than 7 dB. Both these issues cannot be solved by improving the design, since it is already quasi-optimum in this sense. Hence, the only way to lower the insertion loss and reduce the required *B* biasing field is to use graphene with higher mobility^4^, such as graphene encapsulated in hexagonal boron nitride with room temperature mobilities in the order of 100,000 cm^2^ V^−1^ s^−1^. With a mobility of 40,000 cm^2^ V^−1^ s^−1^ and a biasing field of 1 T (easily generated by rare earths permanent magnets) the insertion loss for perfect isolation would be as low as 0.3 dB according to the upper bound^4^, paving the way to commercially relevant devices. These considerations are independent of the carrier density^4^ and, even if high mobility is available only for lower carrier density, the design can be adapted using a larger number of graphene layers.

## Methods

### Fabrication process

The reflective substrate has been fabricated starting with an silicon on insulator wafer having a high resistivity silicon device layer with thickness 10 μm a handle layer of 500 μm separated by an SiO_2_ box layer of 4 μm. Platinum (200 nm) was evaporated on the top side of the silicon on insulator wafer and then it was bonded using parylene to a support pyrex wafer. The handle and box layers were then ground and etched leaving the device layer exposed. Three layers of graphene were subsequently transferred on the substrate keeping the PMMA support film between each layer. More details about fabrication and measurements are available in the Supplementary Note 1.

### Model and notation

Reflection coefficients and derived quantities (insertion loss and modulation depth) are defined in terms of electric fields, and consequently the corresponding decibel unit is defined as 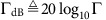.

## 

## Additional information

**How to cite this article:** Tamagnone, M. *et al*. Near optimal graphene terahertz non-reciprocal isolator. *Nat. Commun.* 7:11216 doi: 10.1038/ncomms11216 (2016).

## Supplementary Material

Supplementary Information Supplementary Figures 1-5, Supplementary Notes 1-4 and Supplementary References
