# Supplementary material for: Near optimal graphene terahertz non-reciprocal isolator
Source: Nat Commun. 2016 Apr 6;7:11216. doi: 10.1038/ncomms11216 (PMC4823866; doi:10.1038/ncomms11216)
Supplement: Supplementary Information — Supplementary Figures 1-5, Supplementary Notes 1-4 and Supplementary References [file ncomms11216-s1.pdf]

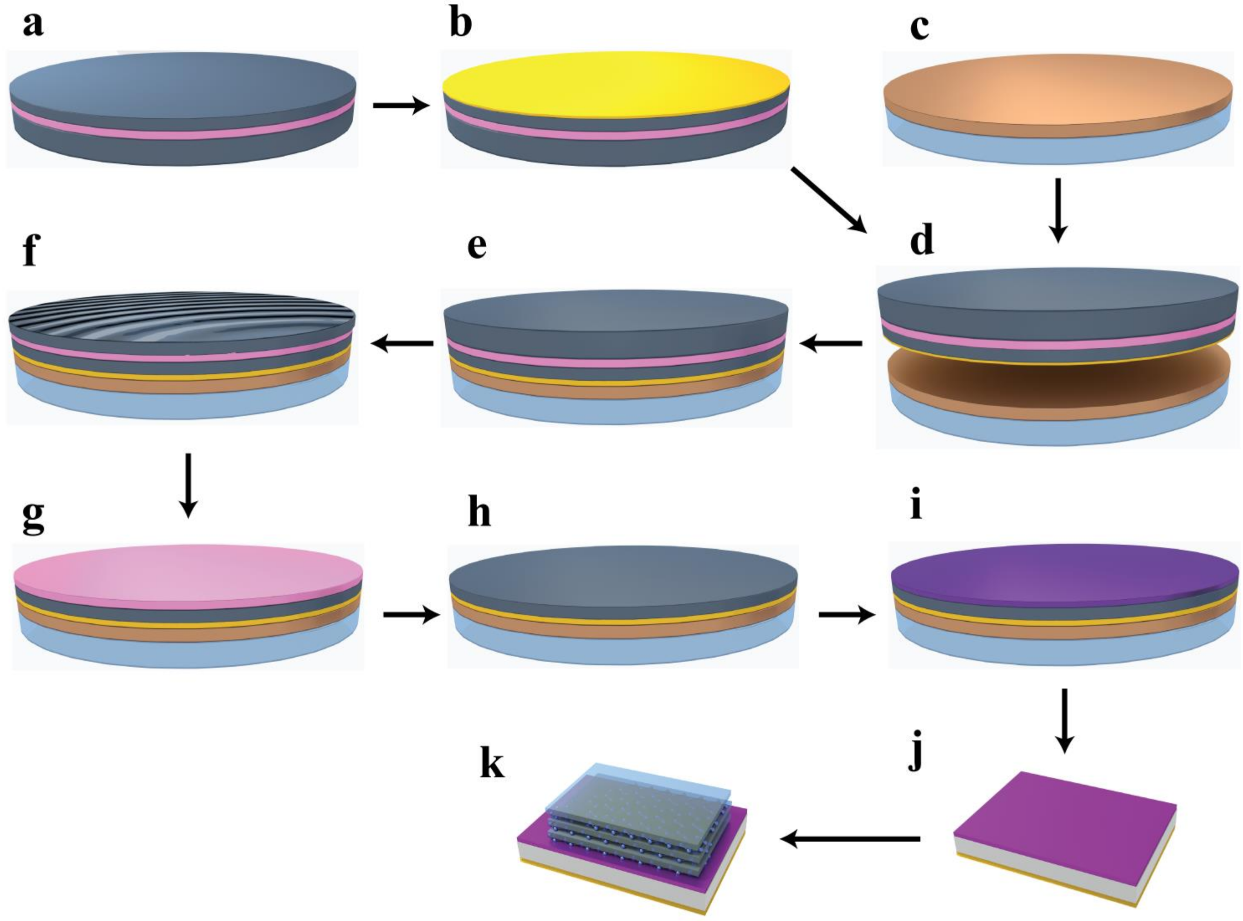

Supplementary Figure 1 | Fabrication process flow. The main steps of the fabrication of the isolator are represented. **a,** initial SOI wafer. **b,** evaporation of the Pt ground plane. **c,** silanization and parylene coating of the pyrex glass. **d-e,** parylene-based wafer bonding. **f,** partial grinding of the SOI silicon handle wafer. **g,** dry etching of the remaining part of the handle wafer. **h,** dry etching of the SiO_2 box layer. **i,** ALD deposition of the Al_2O_3 layer. **j,** dicing into 12 mm square chips. **k,** graphene + PMMA transfer.

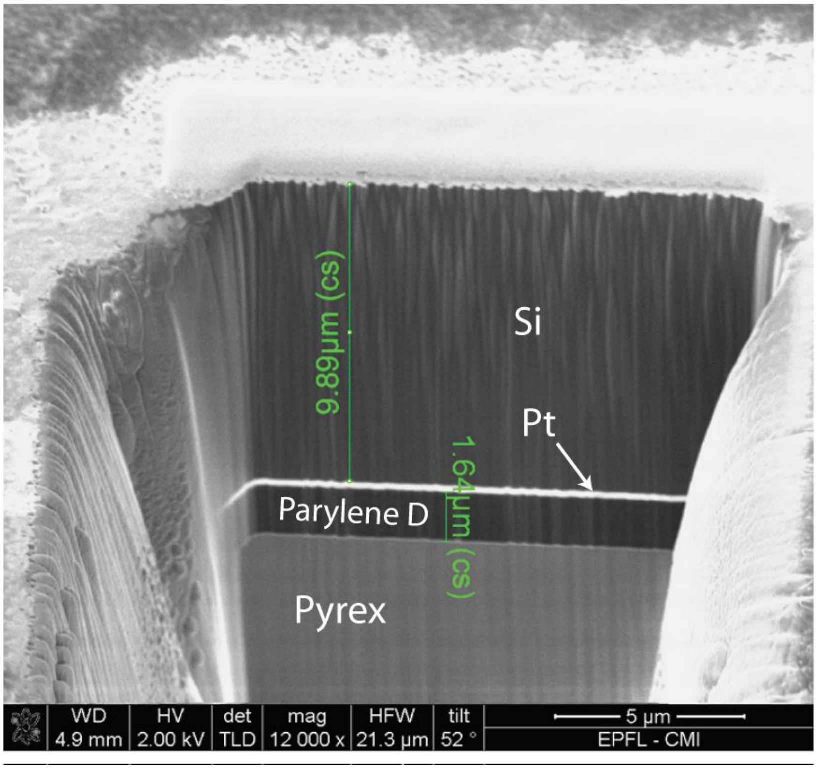

Supplementary Figure 2 | FIB section of the device. The cross section confirms the thickness of the silicon layer (expected to be 10 µm). Because this is a destructive analysis, it has been performed on another chip from the same wafer, hence no graphene-PMMA stack is visible in this picture.

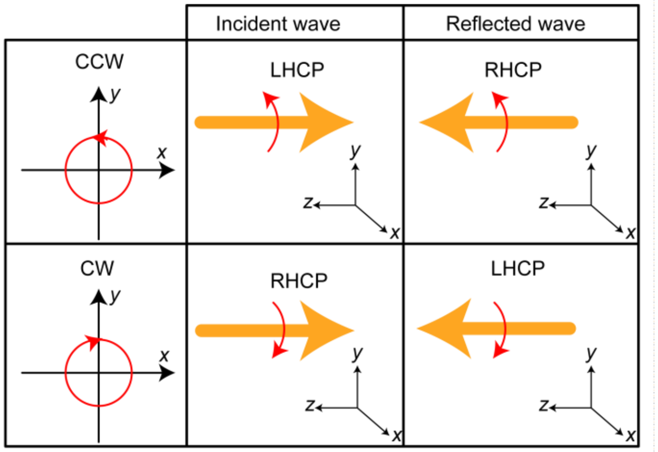

Supplementary Figure 3 | Wave handedness. Handedness of the propagation as a function of wave direction for the CW and CCW cases.

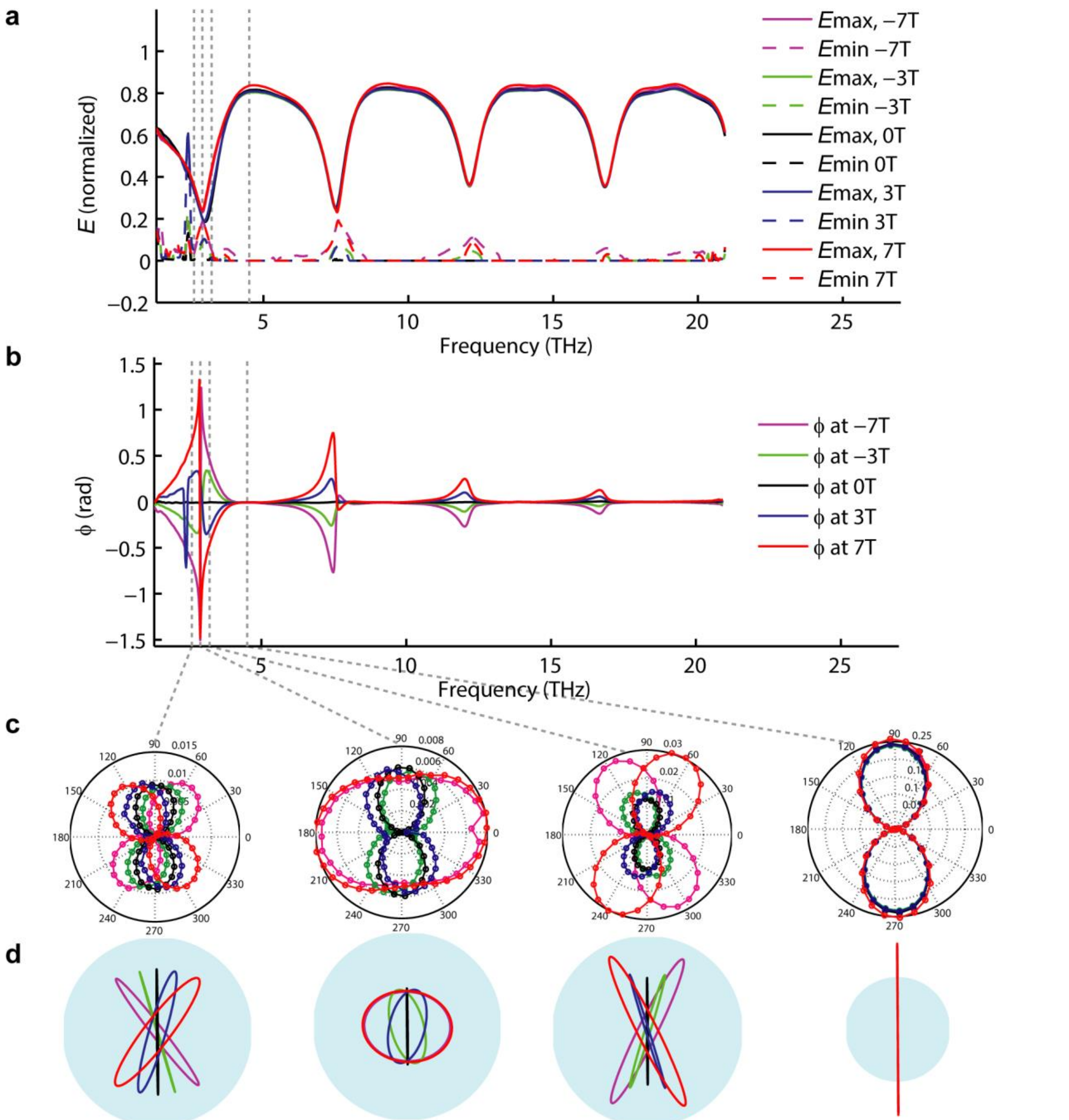

Supplementary Figure 4 | Full device characterization. a,b, polarization parameters. **c,** raw power data for the five magnetic fields values at frequencies 2.6 THz, 2.9 THz, 3.2 THz, 4.5 THz. **d,** corresponding fitted polarization plot (the reference light blue circle has a radius of 0.5. The performance of the device is shown, in the whole measured band up to 20 THz, in panels a and b. There is a striking difference between the behaviour in magnetic field at different frequencies: for f = 4.5 THz, the light stay polarized linearly with no noticeable influence of the magnetic field. For f = 2.9 THz on the contrary, a strong modification of the ellipticity of the light takes place. This variation is also accompanied by a rotation of the light polarization.

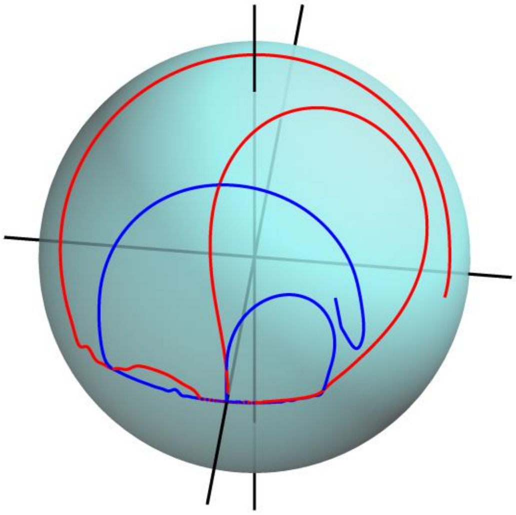

Supplementary Figure 5 | Poincaré sphere for different magnetic fields. The measured for *B* = 7 T (red) and *B* = 3 T (blue) are plotted and compared. This plot represents the polarization state of the reflected light when the incident light is linearly polarized. Ideal isolation is reached if the line passes through the north pole. In fact the incident linear polarization can be split in two counter-rotating circular polarized components, and for ideal isolation one of the two is absorbed and the other reflected. For *B* = 3 T the line is not sufficiently close to the north pole of the sphere, and hence the isolation is very poor. Note that the measure can exchange the CW and CCW case and hence all the data is projected in the north hemisphere.

Supplementary Note 1: Fabrication

Supplementary Figure 1 shows the complete fabrication process flow for the proposed isolator. The most challenging aspect of the fabrication of the proposed device is the creation of a low loss silicon layer having a thickness of 10 µm with a back metallization providing good reflectivity for terahertz waves. To this aim, SOI (silicon on insulator) wafers having a device layer with the required specifications (thickness 10 µm, resistivity >10,000 Ω·cm) were purchased from a commercial vendor (ULTRASIL corporation). The handle layer was 500 µm thick, while the box SiO_2 layer had a thickness of 750 nm. 200 nm of Pt were evaporated (using 10 nm of Cr for adhesion) on the device layer, forming the metallic reflector. The metallized face of the SOI wafer was subsequently bonded to a support pyrex wafer (500 µm) using parylene bonding[1]. The pyrex wafer, chosen for its thermal expansion coefficient very close to the silicon one, works as a support for the thin device layer for the subsequent fabrication steps. The bonding process consists in a piranha cleaning of the pyrex wafer followed by silanization and parylene-D coating (1.64 µm of measured thickness). The two substrates were bonded at 390 °C (10 °C above parylene-D melting point) under vacuum using a tool pressure of 1000 mbar in a Suss SB6 vacuum bonder for a total time of 40 minutes. The handle layer of the bonded SOI wafer was then grinded for a total depth of 400 µm, and the remaining 100 µm of silicon were etched using a deep reactive ion etching (DRIE) process very selective over the SiO_2, to prevent damages to the device layer. The SiO_2 box layer was then removed using a dry etching process with high selectivity over silicon. Using atomic layer deposition (ALD) 72 nm of Al_2O_3 were then deposited on the device layer as final substrate for graphene. After dicing the wafer, commercial CVD monolayer graphene provided by Graphenea Inc. was transferred on the chip using a support PMMA layer of 60 nm. Three graphene monolayers were transferred on the chip without removing the PMMA, thus avoiding inter-layer coupling and preserving the conductivity properties of monolayer graphene. Supplementary Figure 2 shows a section of the isolator before graphene transfer obtained with a focused ion beam (FIB) setup. The image confirms that the bonding is successful and that the thickness of the layers is the expected one.

Supplementary Note 2: Device Model

This section illustrates a model for the proposed isolator, which can be easily derived from Fresnel or Maxwell equations and from simple considerations on the layered structure of the device[2]. In the following, bold quantities are vectors, and underlined quantities are matrices or, equivalently, second-order tensors. When vectors are expressed in bold, they are considered column vectors, and row vectors are always explicitly expressed as the transposition of a column vector. For phasors, the root mean square convention is used, and the engineering time harmonic convention ($e^{+j\omega t}$) is used in this work, so that, e.g., inductors have a positive imaginary impedance. All physical quantities are represented in SI units. All the equations reported from other references are adapted to follow this standard. Reflection coefficients and derived quantities (insertion loss, modulation depth) are defined in terms of electric fields, and consequently, when expressed in decibels, the corresponding decibel unit is defined as $\Gamma_{\mathrm{dB}} \triangleq 20\log_{10}|\Gamma|$.

First we approximate the incident and reflected beams as plane waves propagating normally with respect to the device. The approximation is motivated by the very small angle of the beams with respect to the normal (approximately 8 degrees) and by the spot diameter (in the order of millimetres) much larger than the wavelength of interest. Under these assumptions, each dielectric layer supports a

progressive and a regressive wave (i.e. propagating along the z direction or the $-z$ direction respectively as shown in Figure 1b) having arbitrary polarizations in the xy plane.

Dielectric layers (vacuum, PMMA and silicon) are assumed to be isotropic and with unitary relative magnetic permeability. Relative dielectric permittivities of 11.66 and 2.4 are used for silicon and PMMA respectively. The layers below the platinum reflector are not relevant because only a negligible residue of the incident wave propagates beyond the reflector. The latter has to be modelled carefully to include losses in the Terahertz region. A collisional plasma model has been used, which allows to express the equivalent relative permittivity of platinum as[3]:

$$\varepsilon_{\mathrm{r}} = 1 - \frac{{\omega_{\mathrm{p}}}^2}{\omega^2 + j\nu\omega} \tag{1}$$

Where $\omega_{\mathrm{p}} = 7791$ Trad/s is the plasma frequency and $\nu = 56$ THz is the collision rate[3] . Graphene is represented by a gyrotropic conductivity tensor $\underline{\sigma}$. For graphene under magnetostatic bias, it takes the following form[4]:

$$\underline{\sigma} = \begin{pmatrix} \sigma_{\mathrm{d}} & \sigma_{\mathrm{o}} \\ -\sigma_{\mathrm{o}} & \sigma_{\mathrm{d}} \end{pmatrix} \tag{2}$$

The diagonal and off-diagonal conductivities are given by the Kubo formula[4]:

$$\begin{aligned}\sigma_{\mathrm{d}} = \frac{jq_{\mathrm{e}}^2 v_{\mathrm{f}}^2 |q_{\mathrm{e}}B|\hbar(\omega - j\tau^{-1})}{\pi} \sum_{n=0}^{\infty} \Bigg\{ & \frac{1}{M_{n+1} - M_n} \cdot \frac{f(M_n) - f(M_{n+1}) + f(-M_{n+1}) - f(-M_n)}{(M_{n+1} - M_n)^2 - (\omega - j\tau^{-1})^2\hbar^2} \\ & + \frac{1}{M_{n+1} + M_n} \cdot \frac{f(-M_n) - f(M_{n+1}) + f(-M_{n+1}) - f(M_n)}{(M_{n+1} + M_n)^2 - (\omega - j\tau^{-1})^2\hbar^2} \Bigg\}\end{aligned} \tag{3}$$

$$\begin{aligned}\sigma_{\mathrm{o}} = -\frac{q_{\mathrm{e}}^3 v_{\mathrm{f}}^2 B}{\pi} \sum_{n=0}^{\infty} & \{f(M_n) - f(M_{n+1}) + f(-M_{n+1}) - f(-M_n)\} \\ & \cdot \left\{ \frac{1}{(M_{n+1} - M_n)^2 - (\omega - j\tau^{-1})^2\hbar^2} + \frac{1}{(M_{n+1} + M_n)^2 - (\omega - j\tau^{-1})^2\hbar^2} \right\}\end{aligned} \tag{4}$$

$$M_n = \sqrt{2n v_{\mathrm{f}}^2 |q_{\mathrm{e}}B|\hbar} \tag{5}$$

$$f(\varepsilon) = \left(e^{\frac{\varepsilon - \mu_{\mathrm{c}}}{k_{\mathrm{B}}T}} + 1 \right)^{-1} \tag{6}$$

The formula above expresses the dependence of conductivity on graphene parameters: B is the magnetostatic bias, τ is the carrier scattering time, $\omega = 2\pi f$ is the working frequency, T is the temperature, μ_{c} is graphene Fermi level. Remaining quantities are constants: $v_{\mathrm{f}} = 1 \cdot 10^6$m/s is the Fermi velocity in graphene, q_{e} is the elementary charge, $\hbar$ is the reduced Planck's constant, k_{B} is Boltzmann's constant.

Although in scientific literature the conductivity form of Supplementary Equation 2 is sometimes referred to as *anisotropic*, this terminology is in fact incorrect, as the tensor is left invariant by a rotation; the term *gyrotropic* should be used instead. In a general stack including graphene, dielectric and metal layers, the propagation of the electric and magnetic fields can be determined solving the Maxwell-Fresnel equations for the polarization state for all z coordinates. However, as mentioned in the main paper, an important simplification is possible because of the invariance of the conductivity $\underline{\sigma}$ to a rotation in the xy plane. We first note that $\underline{\sigma}$ has the following eigenvalues and eigenvectors:

$$\begin{aligned} \sigma_{\mathrm{CW}} &= \sigma_{\mathrm{d}} + j\sigma_{\mathrm{o}} \qquad \boldsymbol{v}_{\mathbf{CW}} = \frac{\sqrt{2}}{2}\begin{pmatrix} 1 \\ j \end{pmatrix} \\ \sigma_{\mathrm{CCW}} &= \sigma_{\mathrm{d}} - j\sigma_{\mathrm{o}} \qquad \boldsymbol{v}_{\mathbf{CCW}} = \frac{\sqrt{2}}{2}\begin{pmatrix} 1 \\ -j \end{pmatrix} \end{aligned} \tag{7}$$

The eigenvector $\boldsymbol{v}_{\mathbf{CW}}$ represents a vector which is rotating clockwise in the xy plane, while $\boldsymbol{v}_{\mathbf{CCW}}$ rotates counter-clockwise, which is the reason for the subscripts CW and CCW. The propagation of waves in the isolator can then be studied separately for CW and CCW components to completely characterize the response of the system. For a CW wave the graphene has an equivalent scalar conductivity σ_{CW}, and, similarly, for a CCW wave it is σ_{CCW}. Importantly, even if the propagation can be split for the clockwise and counter-clockwise cases, it is in general impossible to separate the propagation for left hand circular polarized (LHCP) waves and right hand circular polarized (RHCP) ones. In fact the handedness of the wave is determined by the rotation direction in the xy plane *plus* the propagation direction (progressive or regressive), as illustrated in Figure 1b and Supplementary Figure 3. The final behaviour of the structure can then be determined by solving the Fresnel's (or equivalently Maxwell's) equations in all the layers of the structure using the appropriate interface boundary conditions at each layer. The final result consists in the reflection coefficients of the structure that, following Supplementary Figure 3 convention, can be identified as $\Gamma_{\mathrm{L}\to\mathrm{R}} = \Gamma_{\mathrm{CCW}}$ and $\Gamma_{\mathrm{R}\to\mathrm{L}} = \Gamma_{\mathrm{CW}}$.

The full model presented so far has been used to fit the measured curves in the main paper. However, for design purposes, a simplified version of this model can be obtained neglecting the thin PMMA layers and the losses in the metal (hence considering it as a perfect conductor). The final reflection coefficient for the two cases CW and CCW is then given by this simplified analytical formula.

$$\Gamma_{\mathrm{CW,CCW}} = \frac{1 - \eta N \sigma_{\mathrm{CW,CCW}} + jn\cot(nk_0 d)}{1 + \eta N \sigma_{\mathrm{CW,CCW}} - jn\cot(nk_0 d)} \tag{8}$$

where N is the number of graphene layers, n is the refractive index of silicon ($= \sqrt{11.66}$), d represents its thickness, k_0 is the wavenumber in vacuum and η is the vacuum impedance.

This approximate model simplifies the design and the interpretation of the system behaviour. The cotangent expresses the presence of Fabry-Perot resonances in the structure. The condition for the isolation is only met at these resonances, as explained in the next section.

Supplementary Note 3: Design of the Isolator

Unilateral reflection (and hence isolation) can be achieved with a proper choice of N and d. In the following we provide two design rules that ensure this result. First, the magnetic field induced splitting in the equivalent conductivity can be used to create a near-perfect absorption in the CW case *only*, while

maximizing the reflection in the CCW case. This can be achieved if the condition $\Gamma_{\mathrm{CW}} = 0$ is satisfied, namely:

$$1 - \eta N \sigma_{\mathrm{CW}} + jn \cot(n k_0 d) = 0 \tag{9}$$

Because η and n are real, taking the real part of Supplementary Equation 9 the first design rule can be expressed as:

$$N \cdot \mathrm{Re}(\sigma_{\mathrm{CW}}) = \eta^{-1} \tag{10}$$

After characterizing graphene at terahertz frequencies it was determined that for $N = 3$ the first design rule was satisfied for a field approximately equal to 7T. This fact is illustrated in Figure 1c which shows a real part for σ_{CW} of 0.87 mS, very close to $\eta^{-1}/3 \cong$ 0.88 mS. Second, taking the imaginary part of Supplementary Equation 9 we can determine a second design rule, namely:

$$N \cdot \mathrm{Im}(\sigma_{\mathrm{CW}}) = n\eta^{-1} \cot(\omega n d/c) \tag{11}$$

which can be used to determine d. Targeting a working frequency of 3 THz, we determined $d = 10\mu m$. The conductivities of graphene σ_{CW} and σ_{CCW} satisfy the relationship $\sigma_{\mathrm{CCW}} = \sigma_{\mathrm{CW}} - 2j\sigma_{\mathrm{o}}$. This difference creates a reflection in the CCW case:

$$\Gamma_{\mathrm{CCW}} = \frac{\eta N j \sigma_{\mathrm{o}}}{1 - \eta N j \sigma_{\mathrm{o}}} \tag{12}$$

Clearly, the larger is B (and hence σ_{o}) the larger is the reflection, which implies that the isolator has lower insertion losses for high magnetostatic bias.

The working principle of the device can also be explained in terms of Fabry-Perot resonances, as the cotangent periodicity found in Supplementary Equation 11 predicts. Because of this, a second working point around 7.5 THz is possible and it is confirmed by the measurements. However, because of the frequency dispersion of graphene conductivity, in this second working point the design rules are actually satisfied for the CCW case, inverting the direction of the isolator. For higher frequencies graphene conductivity is too small, preventing other working points, which however could be targeted increasing the number of layers.

Supplementary Note 4: Measurement Setup and Data Elaboration

The magneto-optical setup (Figure 2a) measures the sample in reflection (reflection angle of approximately 8 degrees) and it comprises two linear polarizers actuated by a rotary stage, one on the source path and the other on the detector path. Although the isolator works for circularly polarized waves, it is possible to characterize it using linear polarizers following the procedure detailed below. A reference mirror has also been measured together with the isolator to provide an accurate calibration of the system. Being both the isolator and the mirror invariant to a rotation, it is only meaningful to rotate one of the polarizers, while the other can be left at a fixed position. In this set of measurements the polarizer in the detector's path has been kept fix, and the measure has been performed for $N_\theta = 36$ equidistant angles (in the interval $0 - 2\pi$) of the one on the reflection path. The FTIR for N_θ measurements has been performed on the mirror and on the isolator for different magnetostatic fields B. The resulting spectra represent the intensity transmitted from the source to the detector through the isolator and the two polarizers. The data has been subsequently fitted to a polarization ellipse for each

frequency. More specific, for any general elliptical polarization, the power amplitude A depends on the angle of the polarizer accordingly to Malus' law:

$$A(\theta) = A_{\min} + (A_{\max} - A_{\min}) \cos^2(\theta - \phi_0) \tag{13}$$

$A_{\max}$ represents the maximum transmitted power over all the polarizer angles, which occurs when the detector's polarizer is parallel to the major axis of the polarization ellipse. $A_{\min}$ represents instead the minimum transmitted power (i.e. the minor axis cross-polarization). ϕ_0 is the angle of the major axis (Kerr rotation).

The result of fitting $A_{\max}$, $A_{\min}$ and θ_0 for all the frequencies consists then of the following quantities:

$$\begin{array}{ccc} A_{\max}^{\text{ref}}(\omega) & A_{\min}^{\text{ref}}(\omega) & \phi_0^{\text{ref}}(\omega) \\ A_{\max}^{\text{sample}}(\omega) & A_{\min}^{\text{sample}}(\omega) & \phi_0^{\text{sample}}(\omega) \end{array} \tag{14}$$

where the superscript specifies if the data belongs to the reference mirror or to the sample to be characterized.

$A_{\max}^{\text{sample}}(\omega)$ indicates the power received at the detector, and can be used as a reference to compute the transmission properties of the sample. $\phi_0^{\text{ref}}(\omega)$ can instead be used to calibrate the measurement of the polarization rotation introduced by the sample (i.e. the magneto-optic Kerr rotation, MOKE). The complete reflection characterization of the device is then provided by three electric field quantities derived by the power ones:

$$\begin{aligned} E_{\max}^{\text{norm}}(\omega) &= \sqrt{\frac{A_{\max}^{\text{sample}}(\omega)}{A_{\max}^{\text{ref}}(\omega)}} = \frac{E_{\max}(\omega)}{E_{\text{inc}}(\omega)} \\ E_{\min}^{\text{norm}}(\omega) &= \sqrt{\frac{A_{\min}^{\text{sample}}(\omega)}{A_{\max}^{\text{ref}}(\omega)}} = \frac{E_{\min}(\omega)}{E_{\text{inc}}(\omega)} \\ \phi_{\text{Kerr}}(\omega) &= \phi_0^{\text{sample}}(\omega) - \phi_0^{\text{ref}}(\omega) \end{aligned} \tag{15}$$

where $E_{\max}^{\text{norm}}$ and $E_{\min}^{\text{norm}}$ are the major and minor axis of the normalized electric field polarization ellipse respectively and $\phi_{\text{Kerr}}(\omega)$ is the magneto-optical Kerr rotation. Notice that the previous relationships do not use the quantity $A_{\min}^{\text{ref}}$. This is because the reference mirror does not alter the polarization of the beam, so for perfect polarizers we expect:

$$A_{\min}^{\text{ref}}(\omega) = 0 \tag{16}$$

However, in a real setup, the polarizers are not ideal and $A_{\min}^{\text{ref}}$ can be non-zero. In this case its value can be used to remove the effect of non-ideal polarizers from the measures using the following correction on the axial ratio of the elliptic polarization:

$$E_{\min}^{\text{norm}} \leftarrow E_{\min}^{\text{norm}} - E_{\max}^{\text{norm}} \sqrt{\frac{A_{\min}^{\text{ref}}}{A_{\max}^{\text{ref}}}} \tag{17}$$

The proposed polarimetry method has the drawback of not being able to determine the rotation sense of the elliptic polarization, and consequently the LHCP and RHCP might result inverted. It is however very

easy to retrieve this sole missing piece of information from the simulations of the device, which show an excellent agreement with the measurements. Furthermore, it is possible to retrieve the isolation for circularly polarized waves without any additional information. In fact we note that the incident light on the sample, which is linearly polarized, can be thought as the superposition of an LHCP and an RHCP wave having the same amplitude, while the phase difference determines the angle of the linear polarization. In the working frequency band of the isolator, one of these two components is highly attenuated, while the other is reflected with minor loss. Hence the isolation and insertion loss can be determined from $E_{\mathrm{max}}^{\mathrm{norm}}$ and $E_{\mathrm{min}}^{\mathrm{norm}}$ simply noting that:

$$\begin{aligned} E_{\mathrm{max}}^{\mathrm{norm}} &= \frac{|\Gamma_{\mathrm{L}\to\mathrm{R}}| + |\Gamma_{\mathrm{R}\to\mathrm{L}}|}{2} \\ E_{\mathrm{min}}^{\mathrm{norm}} &= \frac{\big||\Gamma_{\mathrm{L}\to\mathrm{R}}| - |\Gamma_{\mathrm{R}\to\mathrm{L}}|\big|}{2} \end{aligned} \tag{18}$$

After some algebraic manipulations we obtain:

$$\begin{aligned} \max(|\Gamma_{\mathrm{L}\to\mathrm{R}}|, |\Gamma_{\mathrm{R}\to\mathrm{L}}|) &= E_{\mathrm{max}}^{\mathrm{norm}} + E_{\mathrm{min}}^{\mathrm{norm}} \\ \min(|\Gamma_{\mathrm{L}\to\mathrm{R}}|, |\Gamma_{\mathrm{R}\to\mathrm{L}}|) &= E_{\mathrm{max}}^{\mathrm{norm}} - E_{\mathrm{min}}^{\mathrm{norm}} \end{aligned} \tag{19}$$

The isolation among the circularly polarized waves is given by definition by the ratio:

$$ISO \triangleq \frac{\max(|\Gamma_{\mathrm{L}\to\mathrm{R}}|, |\Gamma_{\mathrm{R}\to\mathrm{L}}|)}{\min(|\Gamma_{\mathrm{L}\to\mathrm{R}}|, |\Gamma_{\mathrm{R}\to\mathrm{L}}|)} = \frac{E_{\mathrm{max}}^{\mathrm{norm}} + E_{\mathrm{min}}^{\mathrm{norm}}}{E_{\mathrm{max}}^{\mathrm{norm}} - E_{\mathrm{min}}^{\mathrm{norm}}} = \frac{E_{\mathrm{max}} + E_{\mathrm{min}}}{E_{\mathrm{max}} - E_{\mathrm{min}}} \tag{20}$$

While the insertion loss is simply the largest among $|\Gamma_{\mathrm{L}\to\mathrm{R}}|$ and $|\Gamma_{\mathrm{R}\to\mathrm{L}}|$, and hence

$$IL \triangleq \max(|\Gamma_{\mathrm{L}\to\mathrm{R}}|, |\Gamma_{\mathrm{R}\to\mathrm{L}}|) = E_{\mathrm{max}}^{\mathrm{norm}} + E_{\mathrm{min}}^{\mathrm{norm}} = \frac{E_{\mathrm{max}} + E_{\mathrm{min}}}{E_{\mathrm{inc}}} \tag{21}$$

Supplementary References

1. Ciftlik, A. T. & Gijs, M. A. M. in *Solid-State Sensors, Actuators and Microsystems Conference (TRANSDUCERS), 2011 16th International.* 366-369
2. Pozar, D. M. *Microwave Engineering*. Wiley (2005).
3. Tassin, P., Koschny, T., Kafesaki, M. & Soukoulis, C. M. A comparison of graphene, superconductors and metals as conductors for metamaterials and plasmonics. Nat Photon 6, 259-264. (2012).
4. Gusynin, V. P., Sharapov, S. G. & Carbotte, J. P. Magneto-optical conductivity in graphene. *Journal of Physics: Condensed Matter* **19**, 026222 (2007).
